# Quantitative mechanical profiling of 12 ERCP guidewires: Toward evidence-based device selection

**DOI:** 10.1055/a-2840-7390

**Published:** 2026-04-10

**Authors:** Yusuke Saimyo, Reiko Yamada, Takamitsu Tanaka, Kenji Nose, Yasuaki Shimada, Tetsuro Miwata, Minako Urata, Hayato Nakagawa

**Affiliations:** 1 13081 Department of Clinical Engineering, Suzuka University of Medical Science, Suzuka, Japan; 2 220937 Department of Gastroenterology and Hepatology, Mie University Hospital, Tsu, Japan

**Keywords:** Pancreatobiliary (ERCP/PTCD), Endoscopic ultrasonography, Intervention EUS, Quality and logistical aspects

## Abstract

**Background and study aims:**

Numerous guidewires are available for endoscopic retrograde cholangiopancreatography (ERCP) and interventional endoscopic ultrasound (EUS), each with distinct mechanical characteristics. Although individual features such as tip flexibility or shaft stiffness have been studied, no prior research has comprehensively compared multiple guidewires. This study aimed to quantitatively and visually characterize mechanical properties of guidewires using a standardized bench-top evaluation framework.

**Methods:**

Twelve guidewires available in Japan were tested. Seven mechanical parameters were assessed: tip load, maximum tip load, front and rear shaft stiffness, torque performance, whip performance, and radiographic visibility. All tests were conducted under standardized conditions using dedicated equipment. Performance scores were normalized (1–10 scale), and radar charts were generated for visualization.

**Results:**

Guidewires showed wide variability across parameters. Tip load ranged from 0.026 ± 0.029 to 0.114 ± 0.025 N; maximum tip load from 0.180 ± 0.040 to 0.697 ± 0.059 N. Shaft stiffness ranged from 0 to 0.343 ± 0.006 N (front) and 0.057 ± 0.006 to 0.477 ± 0.006 N (rear). Torque deviation ranged from 19.6 ± 16.7° to 41.0 ± 23.8°; whip performance from 13.5 ± 0.6 to 163.4 ± 77.6 °/sec. Radiographic contrast ranged from 292.7 to 1125.4. Values are mean ± SD unless otherwise stated; radiographic contrast was measured once. Radar chart shapes were classified into four types: bowl, rightward, leftward, and upward. Radar chart visualization revealed distinct mechanical profile patterns among guidewires.

**Conclusions:**

This was the first study to provide integrated mechanical profiles of ERCP and EUS guidewires based on standardized bench-top testing. Radar chart visualization offers an intuitive framework for comparing relative mechanical tendencies and may serve as a useful reference for research and education.

## Introduction

A wide range of guidewires is available for endoscopic retrograde cholangiopancreatography (ERCP) and interventional endoscopic ultrasonography (EUS), and several studies have reported their clinical applications 1 2 3 4 5 6 .

In recent years, increasing attention has been directed toward the mechanical properties of guidewires, and several studies have quantitatively evaluated parameters such as tip flexibility, shaft stiffness, lubricity, and torque transmission 7 8 9 . However, most of these investigations have assessed each property in isolation, and few have systematically compared multiple guidewires under standardized experimental conditions.

Consequently, physicians often rely on personal experience or trial and error when selecting guidewires, owing to the lack of objective mechanical data to inform their decisions. Given the increasing complexity of pancreatobiliary endoscopic procedures and the expanding diversity of available guidewires, a comprehensive and reproducible evaluation system is needed. Such an approach would enable objective, data-driven comparisons among devices and assist physicians in rational wire selection.

Unlike previous studies that primarily focused on stiffness or flexibility 7 8 9 , the present study quantitatively analyzes seven mechanical parameters—including torque and whip performance—to provide an integrated mechanical profile of commercially available ERCP guidewires.

This study aimed to conduct a comprehensive quantitative assessment of commercially available ERCP guidewires. We focused on seven mechanical parameters: tip load, maximum tip load, front and rear shaft stiffness, torque performance, whip performance, and radiographic visibility. By integrating these data into radar chart visualizations, we sought to characterize mechanical profiles of each guidewire and to support evidence-based selection in clinical practice.

## Methods

### Study design

This study consisted of two components: 1) a comprehensive mechanical evaluation of 12 commercially available ERCP guidewires; and 2) a functional validation experiment using a bile duct model to assess whether mechanical characteristics were reflected in insertion performance. For the bile duct model validation, four guidewires (VisiGlide2, M-Through FLEX, J-WIRE Prologue, and VENTY) were selected because they exhibited clearly different mechanical tendencies and were considered representative of the major mechanical categories. Both evaluations were conducted under standardized experimental conditions.

### Study materials

**Fig. 1:**
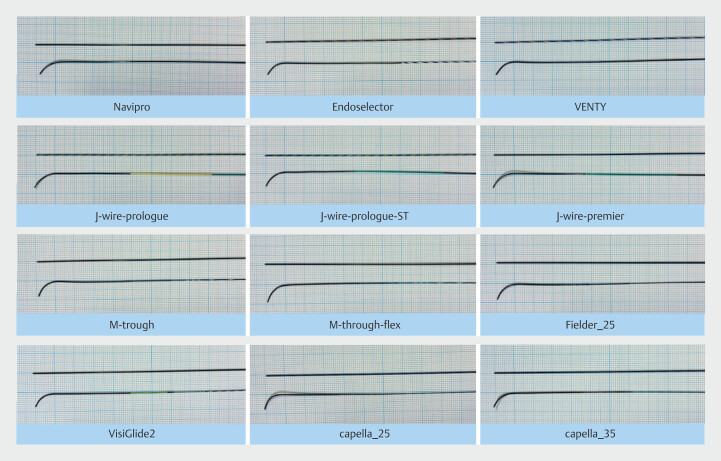
Twelve commercially available ERCP guidewires in Japan evaluated for mechanical performance.

Twelve guidewires commercially available in Japan were selected for evaluation:

VisiGlide2 (Olympus), VENTY, NaviPro, EndoSelector (all Boston Scientific), M-Through, M-Through FLEX, Fielder-25 (all Asahi Intecc), J-WIRE Prologue, J-WIRE Prologue ST, J-WIRE Premier (all J-MIT), CAPELLA25, and CAPELLA35 (both Japan Lifeline) ( Fig. 1 ).

### Evaluation parameters

Seven mechanical properties were selected for assessment, reflecting factors relevant to clinical guidewire performance: tip load, maximum tip load, front-shaft stiffness, rear shaft stiffness, torque performance, whip performance, and radiographic visibility. Each parameter was quantitatively measured using dedicated experimental setups under standardized conditions.

### Measurement protocols

All mechanical measurements were performed using custom-built experimental setups designed for reproducibility. A stepping motor controlled by a MAKER UNO microcontroller board (Cytron Technologies, Malaysia) regulated travel distance and speed of wire advancement. Applied forces were measured using a force gauge (FG-5005, Sato Shouji Inc, Japan). Torque transmission characteristics were assessed with a torque testing system (PT-1950G, Protech Co., Japan).

### Tip load and maximum tip load

**Fig. 2:**
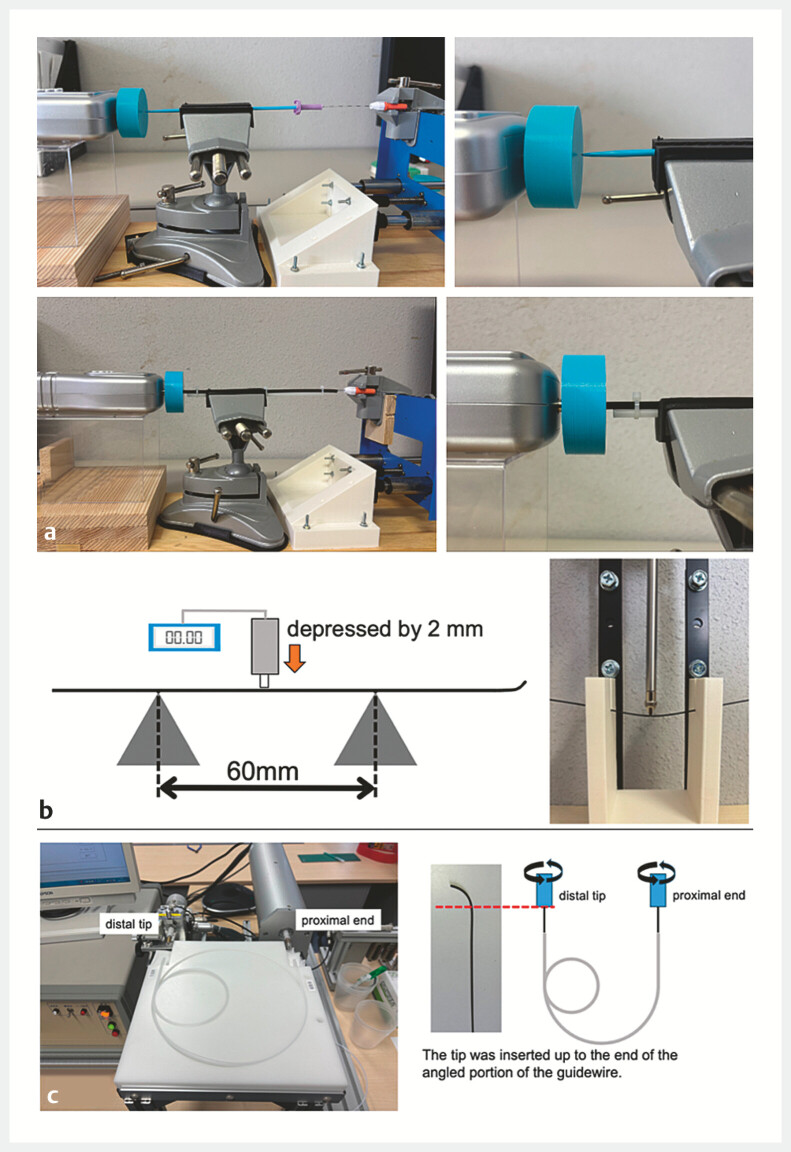
Experimental setups for mechanical property measurements of guidewires. Comprehensive illustration of all measurement setups used in this study. **a** Tip flexibility tests using a force gauge. The tip load was measured in a simulated duct with a 3.0-mm PTFE tube, and the maximum tip load with a 1.0-mm sheath. **b** Shaft stiffness test: Each guidewire was supported horizontally across a 60-mm span, and a 2-mm downward displacement was applied at the center using a force gauge. **c** Rotational response test. The proximal end was rotated at 360°/min for 2 minutes while tracking the distal tip angle over time. This setup was used to calculate torque performance and whip performance.

Two distinct tests were performed to assess tip flexibility. For the Maximum Tip Load Test, each guidewire was inserted into a 1.0-mm inner-diameter sheath and advanced at 0.5 mm/sec toward a force gauge. The highest recorded force was defined as the maximum tip load ( Fig. 2 **a** ). For the Tip Load Test in Simulated Duct, each guidewire was inserted into a 3-mm inner-diameter polytetrafluoroethylene tube and advanced 1 mm at 0.5 mm/sec. The load after 1 mm of advancement was recorded as the tip load ( Fig. 2 **a** ). Each test was repeated three times, and the mean value was used. Lower force values indicate greater flexibility.

### Shaft stiffness

Each guidewire was supported horizontally across a 60-mm span. A downward displacement of 2 mm was applied at the center using a force gauge ( Fig. 2 **b** ). Measurements were taken at two points; 200 mm from the distal tip (rear shaft stiffness) and the proximal endpoint of the hydrophilic coating (front-shaft stiffness). Each measurement was performed three times, and the average value was recorded. Higher values reflect greater stiffness.

### Torque performance

Each guidewire was mounted on a torque transmission test device, with the proximal end rotated at 360° per minute for 2 minutes (measured twice for each wire). Rotational angle of the distal tip was recorded at 90° intervals up to 360°. Mean angular deviation between the proximal and distal ends was calculated. Lower angular deviation values indicate better torque transmission ( Fig. 2 **c** ).

### Whip performance

Whip performance was derived from torque data by calculating the peak rotational speed (°/sec) of the distal tip at 1-second intervals. A higher peak speed indicates more pronounced whip motion.

### Radiographic visibility

Under fluoroscopic imaging, the pixel intensity of each guidewire was measured and compared with background intensity. Fluoroscopic images were obtained using a CUREVISTA fluoroscopy system (Hitachi Medical Systems, Tokyo, Japan) set to 90 kV and 1.7 mA. The difference in mean pixel values was used as the contrast index, with higher values indicating superior radiographic visibility.

### Data normalization and visualization

All mechanical properties were normalized on a 1 to 10 scale based on the range of observed values. For parameters where higher values indicate superior performance (e.g., shaft stiffness, visibility), higher raw values corresponded to higher scores. For torque performance (where lower angular deviation is favorable), lower values were converted to higher scores. Radar charts were constructed for each guidewire to visually represent relative performance across all metrics.

### Bile duct model insertion test

This experiment was not designed to compare guidewire performance or determine superiority, but to provide an exploratory assessment of insertion-related behavior under controlled model conditions.

**Fig. 3:**
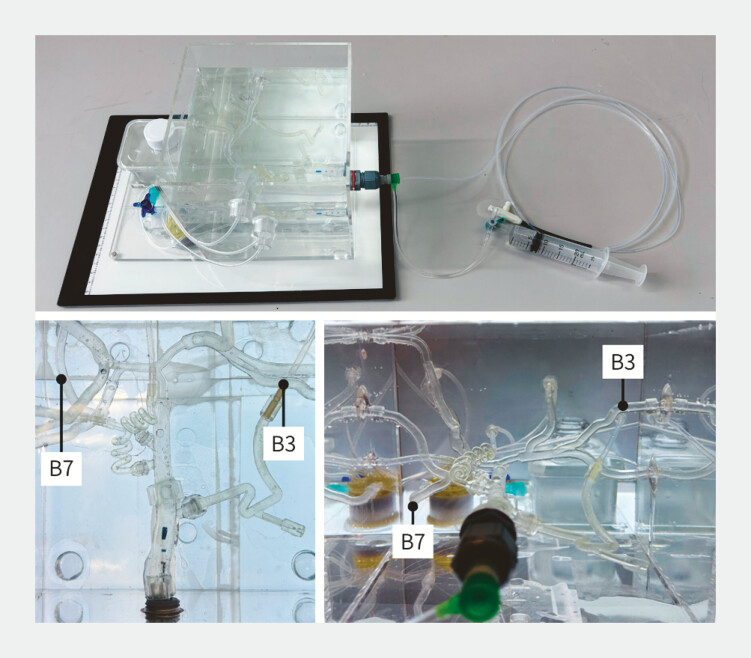
Bile duct model for guidewire insertion test. A water-filled bile duct model was used to assess guidewire insertion performance toward the B3 and B7 branches. A standard cannula was placed at the distal common bile duct, and the guidewire alone was advanced under a Latin-square–based crossover design. Insertion success (within 60 s) and insertion time were recorded for each trial.

To this end, we examined whether the distinctive mechanical tendencies visualized in the radar charts would qualitatively manifest during insertion using a bile duct model. Four guidewires with distinctive mechanical profiles (VisiGlide2, M-Through FLEX, J-WIRE Prologue, and VENTY) were evaluated using a bile duct model ( Fig. 3 ). Bile duct model insertion tests were performed by five endoscopists, each of whom had experience with more than 300 lifetime ERCP procedures. Each guidewire was tested three times for insertion into both the B3 and B7 branches, resulting in six trials per wire for each endoscopist. To minimize learning curve and order effects, a Latin-square–based crossover design was employed, assigning different insertion sequences of the four guidewires to each endoscopist. A standard ERCP cannula (Tandem, Boston Scientific) was placed in the common bile duct of the water-filled model without manipulation. The guidewire alone was advanced from the papillary orifice toward the target branch. The outcome measures were insertion time (seconds) and insertion success. Successful insertion was defined as reaching the target branch within 60 seconds; failure was defined as exceeding this time. Insertion time was recorded using a digital stopwatch.

## Results

All values are presented as mean ± standard deviation (SD), unless otherwise stated. Radiographic visibility was assessed with a single measurement. Differences among guidewires were analyzed using one-way ANOVA followed by Tukey’s post-hoc test, with statistical significance defined as *P* < 0.05.

### Tip flexibility

**Fig. 4:**
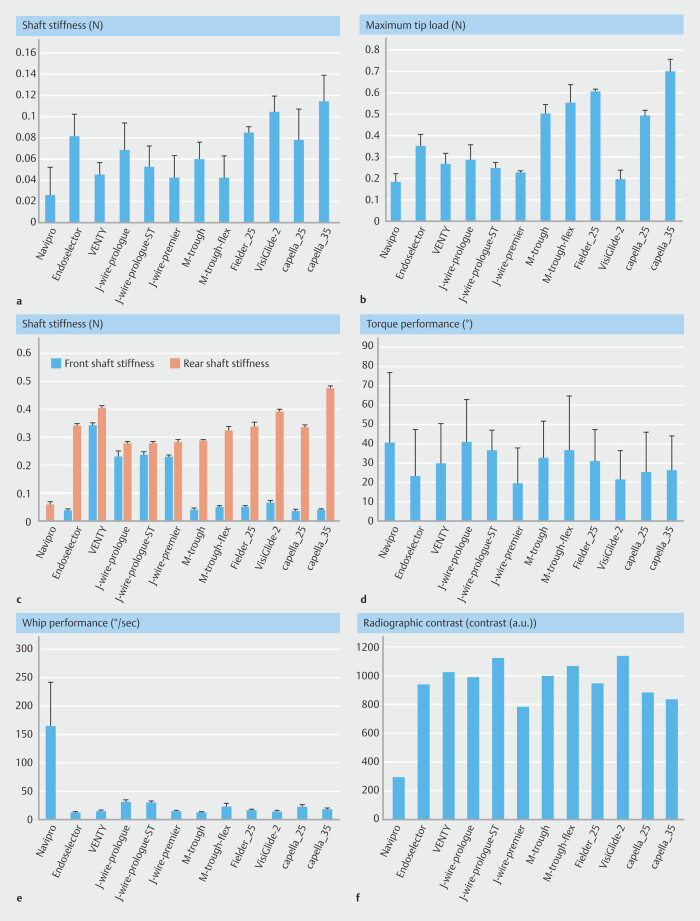
**a** Tip load values (N) measured in a simulated duct using a 3.0 mm inner-diameter PTFE tube. Lower values indicate greater tip flexibility. **b** Maximum tip load values (N) assessed using a 1.0-mm inner-diameter sheath, simulating passage through narrow strictures. Higher values reflect greater force-bearing capacity. **c** Front and rear shaft stiffness (N) of each guidewire. Rear stiffness was measured 200 mm from the distal tip; front stiffness at the proximal end of the hydrophilic coating. **d** Torque performance of each guidewire, expressed as average angular deviation (°) between proximal and distal ends during continuous rotation. Lower values indicate superior torque transmission. Navipro > EndoSelector, VENTY, J-wire-premier, VisiGlide2, Capella25, Capella35. J-wire Prologue > EndoSelector, VENTY, J-wire-premier, VisiGlide2, Capella25, Capella35. J-wire Prologue ST > EndoSelector, J-wire-premier, VisiGlide2, Capella25. M-Through FLEX > EndoSelector, J-wire-premier, VisiGlide2, Capella25. M-Through > VisiGlide2, J-wire-premier. Fielder-25 > J-wire-premier ( *P* < 0.05). **e** Whip performance represented by the peak rotational speed (°/sec) of the distal tip derived from torque testing. Higher values indicate more prominent whip motion. Navipro showed significantly higher whip performance than all other guidewires ( *P* < 0.05). **f** Radiographic contrast (arbitrary units) of guidewires under fluoroscopy. Higher values indicate greater radiopacity. Bars represent mean values; error bars indicate standard deviation (SD), except for radiographic contrast (f), which was measured once. One-way ANOVA revealed no significant differences among the groups in parameters a, b, and c ( *P* > 0.05).

Results of the tip flexibility assessments are shown in Fig. 4 **a** and Fig. 4 **b** .

Tip load values ranged from 0.026 ± 0.029 to 0.114 ± 0.025N ( Fig. 4 **a** ), and maximum tip load values ranged from 0.180 ± 0.04 to 0.697 ± 0.059 N ( Fig. 4 **b** ). A positive correlation was generally observed between tip load and maximum tip load; however, notable exceptions were observed. For example, VisiGlide2 showed a relatively high tip load but a low maximum tip load. In contrast, M-Through FLEX was a guidewire characterized by both a low tip load and a high maximum tip load.

### Shaft stiffness

Shaft stiffness measurements are summarized in Fig. 4 **c** . Front-shaft stiffness ranged from 0.000 ± 0.000 to 0.343 ± 0.006 N, and rear shaft stiffness from 0.057 ± 0.006 to 0.477 ± 0.006 N.

In all guidewires, rear shaft stiffness exceeded front-shaft stiffness. Degree of difference between front and rear shaft stiffness varied across products, reflecting their structural heterogeneity.

### Torque performance

Fig. 4**d** displays torque performance, assessed by angular deviation between proximal and distal ends during controlled rotation. Mean angular deviation ranged from 19.6 ± 16.7° to 41.0 ± 23.8°. Lower deviation values indicated more accurate torque transmission. Guidewires such as J-WIRE Premier and VisiGlide2 showed superior torque control.

### Whip performance

Whip performance, derived from peak angular velocity of the distal tip, is presented in Fig. 4 **e** . Values ranged from 13.5 ± 0.6 to 163.4 ± 77.6 °/sec. Guidewires with lower shaft stiffness, such as NaviPro, demonstrated higher whip activity, while those with higher stiffness tended to suppress whip motion.

### Radiographic visibility

Fig. 4**f** illustrates radiographic visibility of each guidewire under fluoroscopic imaging. Contrast values ranged from 292.7 to 1125.4. Contrast value of NaviPro was markedly lower than that of the other products.

### Comprehensive mechanical profiles

**Fig. 5:**
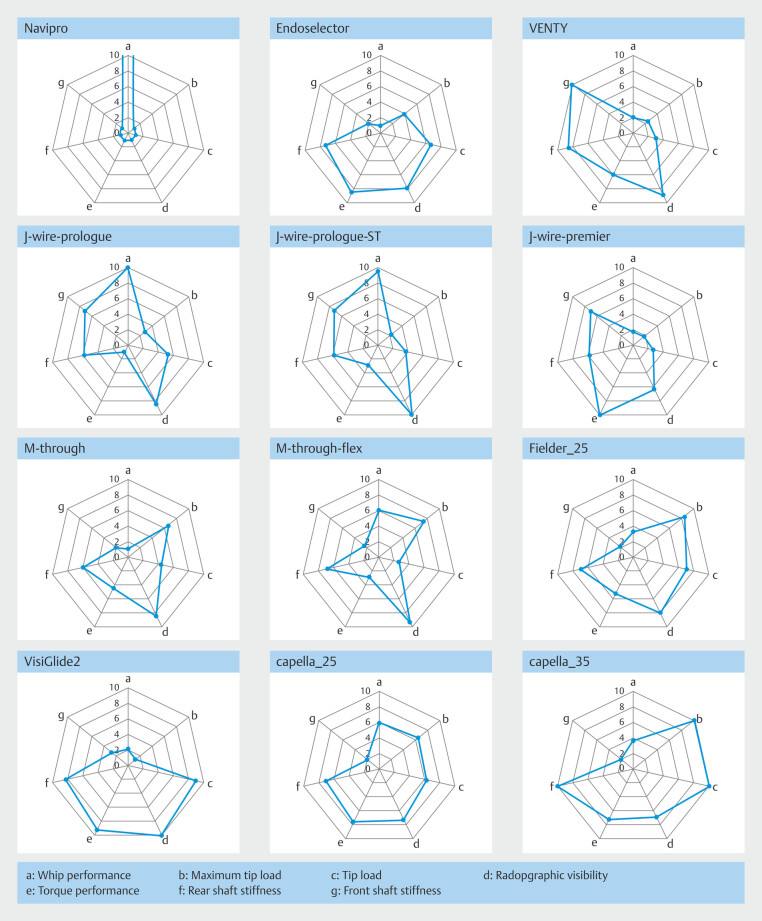
Radar charts summarizing the normalized mechanical performance (scale: 1 to 10) of each guidewire across seven parameters. These charts illustrate distinct mechanical profiles relevant to clinical selection.

To facilitate visual comparison, radar charts were created for each guidewire by integrating all seven mechanical metrics ( Fig. 5 , Table 1 ), with device specifications summarized separately ( Table 2 ).

**Table:** **Table 1** Normalized mechanical performance (scale: 1–10) of each guidewire across seven parameters.

	Front shaft stiffness	Rear shaft stiffness	Whip performance	Torque performance	Maximum tip load	Tip load	Radiographic visibility
Navipro	1.00	1.00	72.58	1.17	1.00	1.00	1.00
Endoselector	1.96	7.14	1.00	8.41	3.96	6.67	7.87
VENTY	10.00	8.50	2.06	5.88	2.45	3.00	8.77
J-wire-pro	7.03	5.71	10.00	1.00	2.80	5.33	8.41
J-wire-pro-st	7.20	5.71	9.47	2.89	2.16	3.67	9.88
J-wire-pre	6.94	5.79	1.74	10.00	1.75	2.67	6.24
M-Through	2.05	6.00	1.11	4.43	6.57	4.33	8.45
M-Through-flex	2.31	6.71	6.08	2.78	7.45	2.67	9.24
Fielder_25	2.22	7.00	3.33	5.22	8.37	7.00	7.96
VISI-2	2.66	8.21	2.16	9.24	1.23	9.00	10.00
capella_25	1.87	7.00	5.87	7.58	6.46	6.33	7.29
capella_35	2.05	10.00	3.65	7.21	10.00	10.00	6.81
Values were calculated using linear scaling, with minimum and maximum values of each parameter set to 1 and 10, respectively.							

**Table:** **Table 2** Device specifications of evaluated guidewires.

	Diameter (inch)	Tip configuration
Navipro	0.025	Angle-tip
Endoselector	0.025	Angle-tip
VENTY	0.025	Angle-tip
J-wire-pro	0.025	Angle-tip
J-wire-pro-st	0.025	Angle-tip
J-wire-pre	0.025	Angle-tip
M-Through	0.025	Angle-tip
M-Through-flex	0.025	Angle-tip
Fielder_25	0.025	Angle-tip
VISI-2	0.025	Angle-tip
capella_25	0.025	Angle-tip
capella_35	0.035	Angle-tip

These visualizations highlighted distinctive performance profiles and allowed categorization of guidewires into four radar chart-based types: Bowl (general-purpose), Rightward (stricture-penetrating), Leftward (delivery-optimized), and Upward (whip-oriented).

Because clinical desirability of higher or lower values differs depending on the parameter (e.g., lower tip load may reduce perforation risk, whereas higher maximum tip load may facilitate stricture passage), we did not regard higher values as universally favorable. Instead, all parameters were normalized to a relative 1 to10 scale to visualize inter-product differences, without directly implying superiority or inferiority.

Parameters plotted on the radar charts were defined as follows: 1) higher tip load and maximum tip load indicate a greater force transmitted to the tip; 2) higher whip performance values represent greater whip motion amplitude, meaning the tip moves more dynamically; 3) higher torque performance indicates smaller angular deviation between the proximal and distal ends, reflecting better torque transmission;

4) higher front and rear shaft stiffness values correspond to increased shaft rigidity; and 5) higher radiographic visibility indicates easier fluoroscopic identification of the guidewire tip.

The scoring formula used for relative evaluation was as follows:

General parameters (tip load, maximum tip load, front and rear shaft stiffness, radiographic visibility, whip performance):

$$Score=1+9\;\times \;\frac{Measured\;value\;-\;Minimum\;value}{Maximum\;value\;-\;Minimum\;value}$$

To mitigate the disproportionate influence of the whip performance value of NaviPro, which was markedly higher than the others, the second-highest value (J-WIRE Prologue) was used as the maximum reference (set to 10) for scaling.

Torque performance was the only parameter for which clinical desirability could be unambiguously defined, because smaller angular deviation consistently indicates better torque transmission. Therefore, raw values were inversely scaled so that smaller deviations corresponded to higher scores:

$$Score=10-9\;\times \;\frac{Measured\;value\;-\;Minimum\;value}{Maximum\;value\;-\;Minimum\;value}$$

### Bile duct model insertion tests

Insertion times and success rates measured using the bile duct model are summarized in Table 3 . VisiGlide2 achieved a 100% success rate and the shortest passage times. M-Through FLEX showed a 93% success rate but required longer passage time in B3 compared with the other guidewires. J-WIRE Prologue demonstrated a high success rate (90%) and relatively short passage times. VENTY had the lowest success rate (80%) and tended to require longer passage times than the other wires.

**Table:** **Table 3** Insertion success rate and passage time in a bile duct model for four guidewires with distinctive radar-chart mechanical profiles.

Guidewire	Insertion success (%)			Insertion time: sec (± SD)	
	Total	B3	B7	B3	B7
VisiGlide2	30/30 (100)	15 (100)	15 (100)	21.5 (± 12.6)	9.5 (± 3.9)
M-Through FLEX	28/30 (93)	13 (87)	15 (100)	34.1 (± 19.5)	16.9 (± 15.1)
J-wire prologue	27/30 (90)	12 (80)	15 (100)	21.2 (± 15.3)	11.6 (± 5.2)
VENTY	24/30 (80)	11 (73)	13 (87)	26.5 (± 15.6)	17.2 (± 13.9)
Values for passage time are shown as mean ± standard deviation (SD) based on successful insertions completed within 60 seconds. Success rates are presented as percentages.					

## Discussion

Although guidewires used in the pancreatobiliary field share a broadly similar structural framework, their mechanical properties can vary substantially, influencing procedure success 7 8 10 . Both high tip flexibility and sufficient shaft stiffness are essential for a safe and efficient procedure 3 4 6 11 12 . However, mechanical data have not yet been fully utilized to guide guidewire selection.

Our study quantitatively evaluated seven parameters in 12 guidewires and integrated them into radar chart visualizations, providing an intuitive and reproducible framework to facilitate comparative understanding of relative mechanical characteristics under standardized conditions.

Tip flexibility, represented by tip load, reflects the stress exerted on the papilla or bile duct wall, whereas maximum tip load indicates resistance to deformation during stricture passage. Clinically, a wire with low tip load and high maximum tip load is optimal for safe cannulation and effective stricture traversal, whereas excessive stiffness may increase perforation risk 3 10 13 14 . Previous studies have reported that a flexible tip facilitates selective cannulation and stricture passage and also reduces risk of perforation 3 4 11 . However, other studies have indicated that high stiffness, a small diameter, and a long hydrophilic tip are important for stricture traversal 10 15 , suggesting that simple tip load alone cannot adequately evaluate stricture passage capability. Therefore, in this study, we simulated strictures and measured this property as the maximum tip load. Although tip load and maximum tip load showed similar trends overall, some guidewires exhibited divergent behavior. In this context, both tip load and maximum tip load represent flexibility of the guidewire tip, but it is considered meaningful to treat them as distinct indicators.

In addition to distal flexibility, shaft stiffness is considered a key determinant of guidewire delivery performance, particularly with respect to force transmission and support along the shaft. An ideal guidewire for device delivery should have high stiffness and be capable of transmitting linear force effectively 6 12 16 . In our study, all guidewires demonstrated higher stiffness in the rear shaft compared with the front shaft, consistent with previous findings 11 . Mechanically, this parameter reflects the ability of the guidewire to transmit linear force, which is considered relevant to support requirements in complex ERCP or interventional EUS. In addition, the difference in stiffness between the rear and front shafts varied among products, and this balance may also contribute to delivery performance.

Another important mechanical characteristic is torque performance. In this study, a mechanically controlled system was employed to measure angular rotation, reducing measurement bias and improving reproducibility.

Conventionally, torque performance is thought to depend on shaft stiffness 8 16 . Guidewires with greater stiffness generally transmitted torque more efficiently under the experimental setup, which may facilitate directional responsiveness during guidewire manipulation.

Whip motion—sudden tip rotation following torque buildup—was inversely related to torque performance. In this study, whip motion was evaluated as a measurable characteristic using peak rotational speed of the guidewire tip over time, offering an objective and reproducible assessment method. We defined whip motion as a sudden tip rotation following a buildup of torque from the handle side. Although whip motion is generally considered undesirable, under controlled conditions, it may influence directional behavior during guidewire manipulation, from a mechanical perspective. Understanding whip characteristics may contribute to mechanical interpretation of guidewire behavior during selective cannulation, although its clinical usefulness requires further validation.

Radiographic visibility was evaluated as a basic imaging characteristic under fluoroscopy. All guidewires demonstrated sufficient visibility to allow clear visualization during simulated device manipulation, even in deep or overlapping biliary segments.

**Fig. 6:**
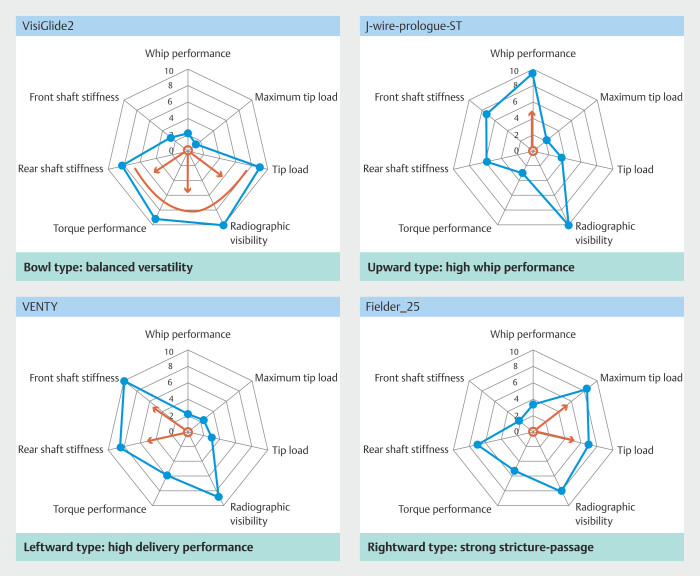
Classification of guidewires based on radar chart patterns. Radar charts of 12 guidewires were grouped into four types: Bowl type (balanced versatility), Rightward type (strong stricture penetration), Leftward type (high delivery support), and Upward type (high whip performance). Each pattern reflects distinct mechanical and clinical characteristics.

Based on the radar chart profiles, guidewires were further categorized into four radar chart-based types according to their characteristic mechanical tendencies ( Fig. 6 ): 1) bowl type, characterized by balanced mechanical versatility (e.g., VisiGlide2, EndoSelector, Fielder-25); 2) rightward type, characterized by strong stricture-passage-related mechanical tendencies (e.g., M-Through, M-Through FLEX, Fielder-25); 3) leftward type, characterized by high shaft stiffness and strong support-related properties (e.g., VENTY, J-WIRE Premier); and 4) upward type, characterized by high whip performance and flexibility (e.g., NaviPro, J-WIRE Prologue ST, J-WIRE Prologue).

This simplified mechanical classification provides a practical reference for understanding guidewires according to procedural objectives and may also serve as an educational framework for trainees ( Table 4 ). To validate this classification, we examined whether the mechanical profiles obtained from radar charts were consistent with known clinical characteristics of representative guidewires.

**Table:** **Table 4** Radar chart-based classification, key mechanical characteristics, and expected clinical applications of the 12 guidewires evaluated.

Guidewire	Type	Characteristic features on radar chart	Mechanically plausible procedural contexts
VisiGlide2	Bowl type	High torque performance and shaft stiffness.	General-purpose manipulation with balanced torque and support.
EndoSelector	Bowl type	High rear shaft stiffness with favorable torque performance.	General-purpose manipulation with balanced torque and support.
VENTY	Leftward type	Strong support capability resulting from high shaft stiffness.	Situations requiring strong shaft support and pushability.
NaviPro	Upward type	High flexibility with outstanding whip performance.	Highly flexible, whip-dominant navigation in tortuous or highly angulated pathways.
J-WIRE Prologue	Upward type	High flexibility with moderate whip performance.	Flexible navigation with moderate whip characteristics in angulated pathways.
J-WIRE Prologue ST	Upward type	High flexibility with moderate whip performance.	Flexible navigation with moderate whip characteristics in angulated pathways.
J-WIRE Premier	Leftward type	High torque performance with relatively high shaft stiffness.	Situations requiring strong shaft support, pushability, and high torque transmission.
M-Through	Rightward type	High maximum tip load with moderate torque performance.	Traversal of resistant or narrowed segments.
M-Through FLEX	Rightward type	Balanced profile of low tip load and high maximum tip load.	Efficient traversal of resistant or narrowed segments with reduced pushing resistance.
Fielder-25	Bowl+Rightward type	High maximum tip load with moderate torque performance.	Broad applicability across varied mechanical demands, with efficient force transmission in narrowed segments.
CAPELLA25	Bowl+Rightward type	High torque performance with adequate tip flexibility and whip performance.	Broad applicability across varied mechanical demands, enabling general-purpose manipulation with balanced torque and support.
CAPELLA35	Bowl+Rightward type	Very high rear shaft stiffness with high tip load.	Situations requiring strong shaft support and pushability.
Each guidewire was categorized into one of four types (Bowl, Rightward, Leftward, or Upward) based on its overall mechanical profile. The columns describe characteristic radar chart features and their conceptually corresponding procedural contexts inferred from mechanical properties.			

Overall, radar chart analysis revealed distinct mechanical profiles among the 12 guidewires, which were broadly consistent with their reported clinical usage patterns in previous studies.

Bowl-type wires such as VisiGlide2 demonstrated balanced torque transmission and stiffness, consistent with their widely reported versatility throughout ERCP procedures 3 4 .

Rightward-type M-Through FLEX, developed as an improved version of M-Through, demonstrated low tip load and high maximum tip load, consistent with previous reports suggesting safe yet effective stricture traversal 17 18 .

Upward-type wires such as NaviPro exhibited extremely high whip performance and flexibility, in line with their reported use for cystic duct access and ETGBD procedures 19 .

The leftward-type CAPELLA35 exhibited high stiffness and torque performance, which is consistent with its reported use in interventional EUS procedures requiring strong wire support 20 .

Representative guidewires such as VisiGlide2, M-Through FLEX, and NaviPro exhibited mechanical profiles that were broadly consistent with previously reported clinical usage patterns. This consistency supports plausibility and interpretability of the proposed mechanical evaluation framework, rather than serving as direct clinical validation.

### Bile duct model insertion tests

The bile duct model experiment was conducted as an exploratory assessment of insertion behavior. It was not intended to evaluate intrinsic performance of guidewires or to determine superiority or inferiority, but rather, to observe whether the distinctive mechanical tendencies visualized in the radar charts qualitatively appeared during insertion under simplified model conditions. The four selected guidewires exhibited particularly distinctive mechanical profiles on the radar charts: balanced characteristics (VisiGlide2), high tip flexibility combined with high maximum tip load (M-Through FLEX), high tip flexibility with high front-shaft stiffness (VENTY), and high whip performance (J-WIRE Prologue). VisiGlide2 demonstrated high success rates and short passage times, whereas M-Through FLEX required longer advancement time because its high tip flexibility reduced transmission of pushing force. VENTY, characterized by high tip flexibility and high proximal-shaft stiffness, had difficulty adjusting direction and tended to form loops near the hepatic hilum, resulting in the lowest success rate among the four wires. Although J-WIRE Prologue exhibited high whip performance, it achieved high success rates and short passage times in the insertion test. This finding suggests that its high whip performance functioned not as instability but rather as enhanced controllability, enabling rapid directional adjustments within branching anatomy. These results support the concept that the mechanical properties visualized by the radar charts can influence insertion behavior. However, because the bile duct model represents a simplified anatomical environment, it does not evaluate stricture-penetration ability, clinical operability, or the comprehensive performance of guidewires.

### Limitations

This study has several limitations that should be considered when interpreting the results.

#### Reproducibility of the testing environment and its divergence from clinical conditions

All measurements were performed under standardized mechanical conditions. However, these do not fully replicate the complex anatomical structures or clinical variables encountered during actual procedures, such as the shape of the biliary and pancreatic ducts, presence of bile sludge or blood, or variations in procedure angles. Accordingly, the mechanical properties quantified in this study should not be interpreted as direct predictors of clinical performance or procedural success. Rather, they should be regarded as reference indicators of relative mechanical tendencies under controlled conditions.

#### Limitations of the radar chart as a relative evaluation tool

The radar chart provides only relative scores (scaled 1–10) for comparison and does not convey absolute clinical significance. For example, the charts do not offer thresholds related to perforation risk or predict cannulation success rates. Consequently, a lower score does not necessarily indicate inadequate clinical performance, but rather reflects relative differences among guidewires within the tested cohort.

#### Limited product representation

This study was restricted to 12 angle-tip guidewires currently available in the Japanese market. Straight-tip guidewires were not included. Therefore, the findings may not fully represent all guidewire designs, and generalizability of the results may be limited.

#### Limitations of the bile duct model experiment

The bile duct model experiment was conducted in a simplified anatomical setting and involved only five endoscopists. Although a crossover design was employed to reduce order effects, guidewire manipulation is inherently operator-dependent and influenced by individual experience and familiarity with specific devices. Therefore, findings from this model should be interpreted as illustrative observations of insertion-related behavior rather than objective comparisons of guidewire performance.

## Conclusions

In this study, we quantitatively evaluated and comparatively analyzed seven mechanical performance parameters across 12 commercially available guidewires: tip load, maximum tip load, front-shaft stiffness, rear shaft stiffness, torque performance, whip performance, and visibility. These radar chart-based visualizations allowed for intuitive comparisons and highlighted the unique strengths of each guidewire. This reproducible evaluation framework bridges bench testing and mechanical understanding, providing structured reference data that may inform future clinical studies and education.
